# Identification and characterization of karyotype in *Passiflora* hybrids using FISH and GISH

**DOI:** 10.1186/s12863-018-0612-0

**Published:** 2018-04-27

**Authors:** Gonçalo Santos Silva, Margarete Magalhães Souza, Cláusio Antônio Ferreira de Melo, Juan Domingo Urdampilleta, Eliana Regina Forni-Martins

**Affiliations:** 1 Departamento de Ciências Biológicas, Universidade Estadual de Santa Cruz (UESC), Ilhéus, BA Brazil; 2 Instituto Multidisciplinario de Biología Vegetal (IMBIV), CONICET – UNC, Córdoba, Argentina; 3 Departamento de Biologia Vegetal, Instituto de Biologia, Universidade Estadual de Campinas, Campinas, SP Brazil

**Keywords:** CMA_3_ and DAPI banding, FISH, GISH, Interspecific hybrids, Passion flowers

## Abstract

**Background:**

A great interest exists in the production of hybrid plants of the genus *Passiflora* given the beauty and exotic features of its flowers which have ornamental value. Hybrid paternity confirmation is therefore important for assuring germplasm origin, and is typically carried out by molecular marker segregation. The aim of this study was to karyotypically characterize the chromosome heritance patterns of the progeny resultant from a cross of *P. gardneri* and *P. gibertii* using classical cytogenetics, chromosome banding, and molecular cytogenetics.

**Results:**

All analyzed genotypes showed the same diploid chromosome number as the genitor species: *2n* = 18. Classical and CMA_3_ and DAPI staining allowed for chromosome counting and satellite identification (secondary constrictions). Fluorescence in situ hybridization (FISH) and genomic in situ hybridization (GISH) were used to characterize subgenomes by either identifying rDNA-specific genome patterns or parental genomes, respectively.

**Conclusions:**

The heritance of chromosomal markers presenting rDNA sites from each parent for genome identification confirmed that all obtained plants were hybrids. These results will improve breeding programs involving the species of this genus. Apart from confirming hybridization, GISH allowed the visualization of recombination between the homeologous chromosome and the introgression of sequences of interest.

**Electronic supplementary material:**

The online version of this article (10.1186/s12863-018-0612-0) contains supplementary material, which is available to authorized users.

## Background

The genus *Passiflora* L., comprising more than 525 species, is the largest within the family Passifloraceae A.L. de Jussieu ex Kunth [1]. Brazil is an important center of diversity with 137 species [2]. Certain species of the genus *Passiflora* have attracted a large economic interest for food purposes, highlighted by the sour passion fruit (*P. edulis* f. *flavicarpa* O. Deg.) [3], as well as for medicinal purposes [4] and ornamental use [5, 6]. The ornamental plant market has expressed great interest in interspecific hybrids in order to facilitate the production of plants with unique characteristics [5]. Most of the hybrids described yield beautiful flowers and exotic foliage varying in color and shape, an essential feature for ornamentation [7].

*Passiflora* species are widely available in the ornamental plant markets of Europe, Japan, and the USA [2]. However, the ornamental potential of *Passiflora* species remains practically unexplored in Brazil, although the location of Brazil in the tropical zone provides favorable climatic conditions for its cultivation [6]. *Passiflora* breeding programs with ornamental intentions have recently gained prominence in Brazil, attempting to produce hybrids possessing unique characteristics, considering the edaphoclimatic conditions of the country [8].

The production of *Passiflora* hybrids for ornamental purposes started a long time ago, yet the genomic and cytogenetic characterization of the generated hybrids is not well explored. Studies verifying the genetic and genomic compatibility of these hybrids and what factors can affect their fertility are therefore necessary. Hybrid identification can be carried out using different techniques, ranging from simple and low-cost options using morphological characteristics [9] to protocols employing molecular markers such as Random Amplified Polymorphic DNA (RAPD), Simple Sequence Repeat (SSR), Amplified Fragment Length Polymorphism (AFLP), and expressed sequence tags (ESTs) [10]. The use of cytogenetic data also offers significant results in hybrid analysis, with conventional and molecular cytogenetics providing a variety of chromosomal characteristics [11]. Chromosomal markers are a useful tool for identifying hybrids and allow the observation of the stability of hybrids produced in breeding programs [12, 13].

Molecular cytogenetic techniques, such as fluorescence in situ hybridization (FISH), are useful for paternity confirmation in hybrids. In particular, specific chromosomes with different marks may be useful, such as the 45S and 5S ribosomal DNA probes (rDNA). Chromosomes presenting rDNA sites can be used as markers to identify the genomes of the hybrid genitor species [14]. In addition, marker chromosomes can aid the observation of karyotype stability during the production of neo-hybrids, improving breeding programs. Another technique which has been widely used for hybrid identification is genomic in situ hybridization (GISH), which involves the use of the total genomic DNA from one species as a probe [15], enabling the observation of the respective genomes of each species present in the hybrid, as well as the observation of whether chromosomal recombination is occurring in different generations of hybrid progeny [16, 17].

*Passiflora* hybridization can be confirmed by morphological and molecular markers using techniques such as RAPD [18, 19] and SSR [8], which are more reliable methodologies for paternity confirmation in passion fruit hybrids. Recently, GISH has been used to confirm hybridization within the genus [20] and to analyze chromosomal recombination in RC_1_ hybrids [21]. The use of FISH for checking hybridization in *Passiflora* species has not been reported. However, this technique has been employed within the genus, specifically, using 45S and 5S rDNA probes to characterize some species [22] and somatic hybrids [23].

The aim of this study was to karyotypically characterize the hybrids and their genitors (*Passiflora gardneri* vs. *Passiflora gibertii*) obtained in an ornamental plant breeding program using classical cytogenetics and staining with specific-base fluorochromes. This study also sought to confirm paternity using in situ hybridization, using GISH and FISH to eliminate the hypothesis of self-fertilization and to evaluate genome cytogenetic stability based on chromosome markers.

## Methods

### Plant material

The species *Passiflora gardneri* Mast. (female parent) and *Passiflora gibertii* NE Brown (male parent) were kept in the Active Germplasm Bank (BAG-Passifloras), located on the campus of the State University of Santa Cruz (UESC) in the city of Ilhéus, Bahia (longitude 39 10“W, latitude 14 39”-S, altitude 78 m). Both species were obtained from the Brazilian Agricultural Research Corporation (Embrapa Cerrados), Brasilia, Brazil. The genitor species were selected based on leaf and flower characteristics. *P. gardneri* presents characteristics, including the structure of its flowers as well as an abundant flowering period running from September to March, which elicits the interest of the ornamental plant market. Likewise, *P. gibertii* is attractive because it presents early growth and flowering, and produces up to 30 flowers per day under normal conditions. Additionally, *P. gibertii* presents resistance to premature death and fusariosis, with has caused great damage to Brazilian passion fruit culture. Finally, *P. gibertii* and *P. gardneri* belong to the same infrageneric level (subgenus *Passiflora*, section *Granadillastrum*). The interspecific crossings between *P. gardneri* vs. *P. gibertii* were performed in a greenhouse with temperature ranging from 25 to 30 °C and a relative air humidity of 70-90%. Pre-anthesis flower buds were protected with white paper bags the day prior to artificial pollination. Fruits resulting from hybridization were protected with nylon nets. After the fruits were fully mature, the seeds were propagated. Twenty-five hybrids germinated and were kept in a greenhouse. The hybrids that presented normal growth and flowering, as well as a wide segregation of colors, shapes, and sizes in their floral parts were selected. Eight F_1_ interspecific hybrids (HD15-101, HD15-104, HD15-106, HD15-107, HD15-108, HD15-109, HD15-110, HD15-111) were analyzed.

### Slide preparation

Root tips of approximately 1 cm in length were collected, pre-treated with 0.002 M 8-hydroxyquinoline (8-HQ; Merck) for 1 h at room temperature (RT) and a further 21 h at 8 °C to 10 °C. After being washed twice in distilled water and fixed in Carnoy (anhydrous ethanol (Merck):glacial acetic acid (Merck) [3:1], *v*/v; [24]) for 3 h at RT, the samples were stored at − 20 °C for at least 24 h. For slide preparation, root apices were washed twice in distilled water and incubated in a humidity chamber at 37 °C with 50 μl of 2% cellulase enzyme solution (Sigma) and 20% pectinase (*w*/*v*) (Sigma) for 80 min. The enzymes were then removed using a micropipette, and the root samples were washed again in distilled water and then added 10 μl of 45% acetic acid (Merck). Roots were then macerated using needles under a stereomicroscope, covered with a cover slip, pressed firmly between filter paper, frozen in liquid nitrogen for approximately 6 min to remove the cover slip, and finally air dried. Slide preparations featuring good presentation of cells in metaphase were kept at − 20 °C until the application of cytogenetic techniques.

Conventional cytogenetic staining for establishing chromosome count was performed following the protocol of Guerra and Souza [25] with modifications consisting of the use of 2% Giemsa solution (Merck) for 20-30 min, followed by briefly rinsing the slides in distilled water and air drying. After staining, the slides were mounted with Neo-Mount medium (Merck) and then coverslipped.

### CMA_3_/DA/DAPI chromosome banding

In order to locate heterochromatin rich in GC and AT, slides were aged for 3 days prior to staining. We have used the fluorochromes Chromomycin A_3_ (CMA_3_; Sigma) and 4′-6-Diamidino-2-phenylindole (DAPI; Sigma) to stain GC and AT base pairs, respectively. A combination of the non-fluorescent antibiotic Distamycin (DA; Sigma) and the fluorochrome DAPI (DA/DAPI) favors differential staining by highlighting loci predominantly composed of AT bases. Coloration with CMA_3_/DA/DAPI was performed following the protocol used by Guerra and Souza [25], with an alteration in the CMA_3_ concentration used [26]. Slides were treated with 15 μl CMA_3_ (0.25 mg/ml) for 1 h, then washed with distilled water and dried. Subsequently, 15 μl Distamycin A (0.1 mg/ml) was applied for 30 min, following which slides were washed with distilled water and dried, then treated with 15 μl DAPI (2 mg/ml) for 30 min. Finally, slides were washed with distilled water, dried, mounted using 15 μl of assembly medium glycerol (Sigma)/Mcllvaine (1:1 *v*/v), and coverslipped (20 × 20 mm). Slides were stored a darkened chamber for 3 days before analysis.

### In situ hybridization probes

DNA from both parent species were extracted using the protocol of Doyle and Doyle [27] for the production of in situ hybridization probes. For GISH, *P. gibertii* total genomic DNA was labeled with biotin-16-dUTP (Roche Diagnostics) via nick translation, and *P. gardneri* total genomic DNA was used as blocking DNA. To prepare blocking DNA, genomic DNA was cleaved with a sonicator (Qsonica Q125) in order to obtain bands preferably between 100 and 800 bp. Sonication resulted in the generation of fragments predominantly between 200 and 1000 bp. In order to break the blocking DNA, about 20 μg of genomic DNA in a final volume of 200 μl was cleaved using sonicator (amplitude 40%, alternating pulses of 2 s on and 2 s off, total duration 5 min) [28]. The sizes of the cleaved fragments was checked using electrophoresis in agarose gel (Pronadisa) 2% using a 100 bp ladder marker as a reference (New England Biolabs). Purification of the cleaved genomic DNA was accomplished through the precipitation of nucleic acids by adding 2% of the final sodium acetate volume (Sigma) to 3 M plus 200% of the final volume of anhydrous ethanol (Merck).The mixture was stored at − 20 °C overnight and then centrifuged (Novatecnica 805 NT) for 10 min at 14,000 rpm at 20 °C to isolate the pellet and eliminate the supernatant. The pellet was dried at RT for at least 1 h before being resuspended with ultrapure water to generate a final DNA concentration of 1.1 μg/μL.

For FISH, pTa71 [29] clones (a donation from the Biosystematics Laboratory, Institute of Biology, State University of Campinas, SP, Brazil) were used to obtain probes for 45S rDNA sites, which were labeled with biotin-16-dUTP (Roche Diagnostics). Probes for 5S rDNA sites were obtained via polymerase chain reaction (PCR) using specific primers (5′-GTGCGATCATACCAGRYTAATGCACCGG-3′ and 5′-GAGGTGCAACACGAGGACTTCCCAGGAGG -3′) [22] and labeled with digoxigenin-11-dUTP (Roche Diagnostics). The probes were labeled using nick translation, with a final DNA concentration of 1 μg, following the protocol proposed by the manufacturer.

The 45S and 5S rDNA probes were used for the identification of marker chromosomes, allowing for karyotype characterization and hybrid status verification.

### GISH and FISH

Slides for FISH were treated in accordance with the protocol described by Schwarzacher and Heslop-Harrison [30] and Souza et al. [31] with modifications [20]. Slides with cytological preparations were dried at 37 °C for at least 1 h. Following this, slides were treated with 50 μl of a solution containing 1 mg/ml RNase (Sigma) in 2× SSC (salt, sodium citrate) buffer (0.3 M sodium chloride [Sigma], 0.03 M sodium citrate [Sigma]) and incubated in a humidified chamber 1 h at 37 °C. The slides were the immersed in 2× SSC at RT twice for 5 min each, and then incubated with 50 μl 10 mM hydrochloric acid (HCl; Vetec) for 5 min. Following this, HCl was removed and replaced with 50 μl of pepsin (Sigma) [10 mg pepsin/ml, 10 mM HCl (1:100 *v*/v)] and slides were incubated in a humidified chamber for 20 min at 37 °C. The slides were then washed in 2× SSC at RT twice for 5 min each, immersed in 4% formaldehyde (Sigma) at 4% for 10 min, and then rinsed again in 2× SSC twice for 5 min each. The wash steps were carried out using a shaker platform (Biomixer Mos-1) set at 120 rpm. Cytological preparations were dehydrated in 70% and 95% ethanol for 5 min each. After drying the slides at RT for 30 min, slides were incubated with 15 μl hybridization mix, consisting of 50% formamide (Sigma), 10% dextran sulfate (Sigma), 2× SSC (Sigma), 0.13% sodium dodecyl sulfate (SDS; Bioagency), and the probes. For GISH, we used 33 ng of probe and 3.3 μg of blocking DNA (100×), while for FISH, we used 50 ng of either the 45S or the 5S probes. The hybridization mixture was heated at 75 °C for 10 min in a thermocycler (Eppendorf Mastercycler) and immediately transferred to ice for a minimum incubation of 5 min. Cytological preparations containing the hybridization mixture were denatured in a thermocycler (Techne TC-412) containing a slide adapter at 75 °C for 10 min and incubated overnight at 37 °C in a humidified chamber. After hybridization, slides were immersed in 2× SSC for 5 min at RT to facilitate coverslip removal, moved to a Dubnoff bath (Quimis Q226M2) set at 42 °C, and immersed in 2× SSC for 5 min each, twice in 0.1× SSC for 5 min each, and twice again in 2× SSC for 5 min each. Finally, slides were dipped in 4× SSC containing 0.2% Tween 20 (Sigma) at RT for 5 min and then treated with 50 μl of 5% bovine serum albumin (BSA; Sigma). Biotin-labeled probes were detected by incubating each slide with a 0.7 μl avidin-fluorescein isothiocyanate (FITC; Vector):19.3 μl 5% BSA solution. Digoxigenin-labeled probes were detected by incubating each slide with a 0.7 μl anti-digoxigenin-rhodamine (Roche):19.3 μl 5% BSA solution. All slides containing antibodies were incubated in a humidified chamber for 1 h at 37 °C. Three washes of 5 min each with 4× SSC containing 0.2% Tween 20 were conducted to remove excess antibody. Finally, the slides were briefly immersed in 2× SSC and cytological preparations were mounted and counterstained with Vectashield® Antifade Mounting Medium with DAPI (M-1200). The slides were stored at 8-10 °C until analysis.

### Chromosome Photodocumentation

Metaphases following fluorochrome staining and in situ hybridization were photodocumented using an epifluorescent Olympus BX41 microscope equipped with a 5 MP Olympus DP25 digital camera and DP2-BSW software. CMA_3_ blocks were detected with a U-MWB filter (excitation 450-480 nm/dichroic cutoff 500 nm/emission > 515 nm) and DAPI signal with a U-MWU filter (excitation 330-385 nm/dichroic cutoff 400 nm/emission > 420 nm). Hybridizations detected using avidin-FITC were visualized with a U-MWB filter (excitation 450-480 nm/dichroic cutoff 500 nm/emission > 515 nm), while hybridizations detected using anti-digoxigenin-rhodamine were visualized using a U-MWG filter (excitation 510-550 nm /dichroic cutoff 570 nm/emission > 590 nm). DAPI counterstaining was detected with a U-MWU filter (excitation 330-385 nm/dichroic cutoff 400 nm/emission > 420 nm). Slide images, karyograms, and FITC/DAPI overlays (for GISH) and FITC/rhodamine/DAPI overlays (for 45S and 5S rDNA sites) were processed using Photoshop SC5.

## Results

### Conventional and Fluorochrome staining

**Fig. 1:**
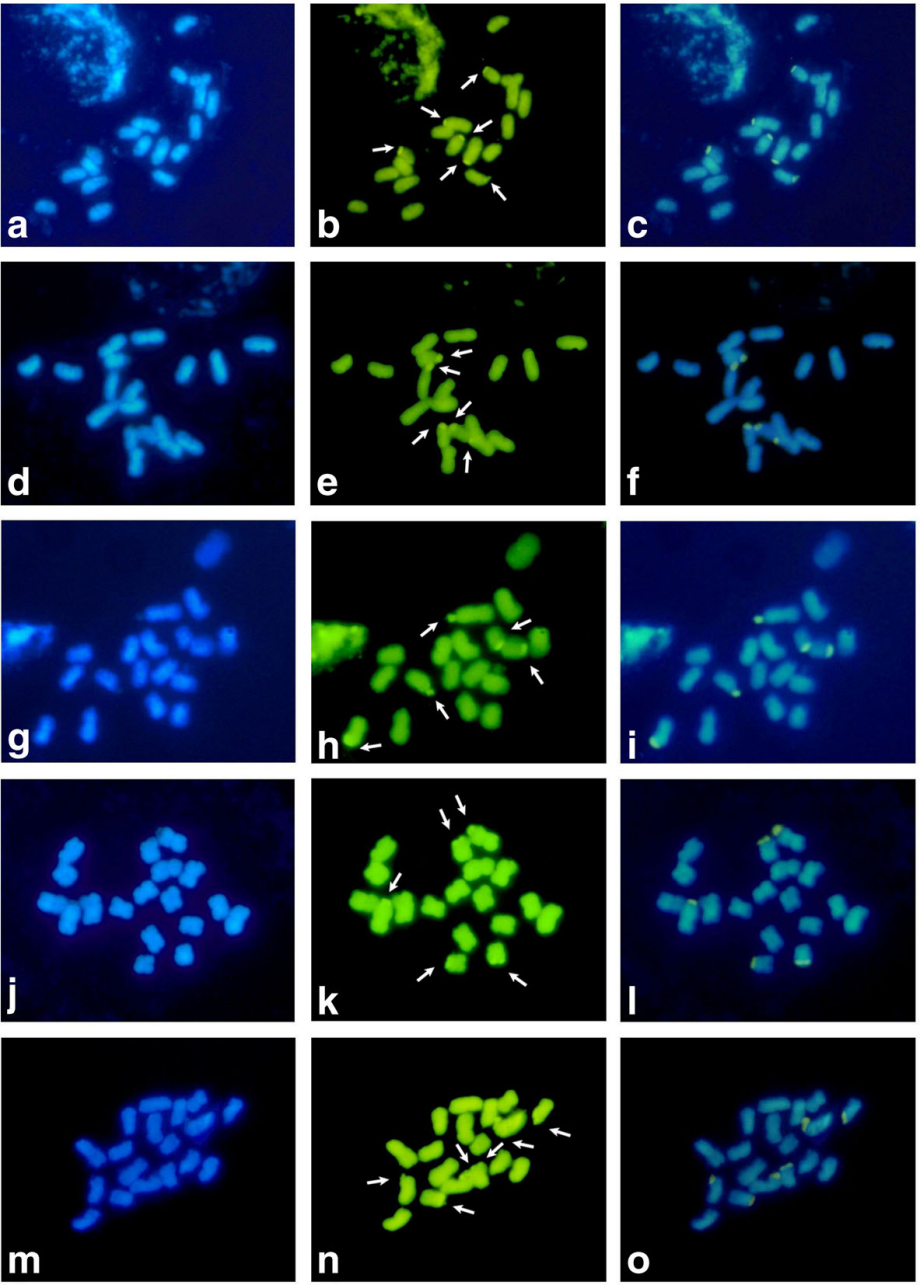
CMA_3_/DA/DAPI banding of mitotic metaphase cells from parents and interspecific hybrids of *Passiflora* HD15 progeny. Staining with DAPI (**a**, **d**, **g**, **j**, **m**), CMA_3_ (**b**, **e**, **h**, **k**, **n**), and CMA_3_/DAPI merged (**c**, **f**, **i**, **l**, **o**). **a**-**c**: *P. gardneri* Mast.; **d**-**f**: *P. gibertii* N. E. Brown; **g**-**i**: HD15-101; **j**-**l**: HD15-104; **m**-**o**: HD15-106. Arrows indicate CMA_3_^+^ blocks. Bar = 10 μm

**Fig. 2:**
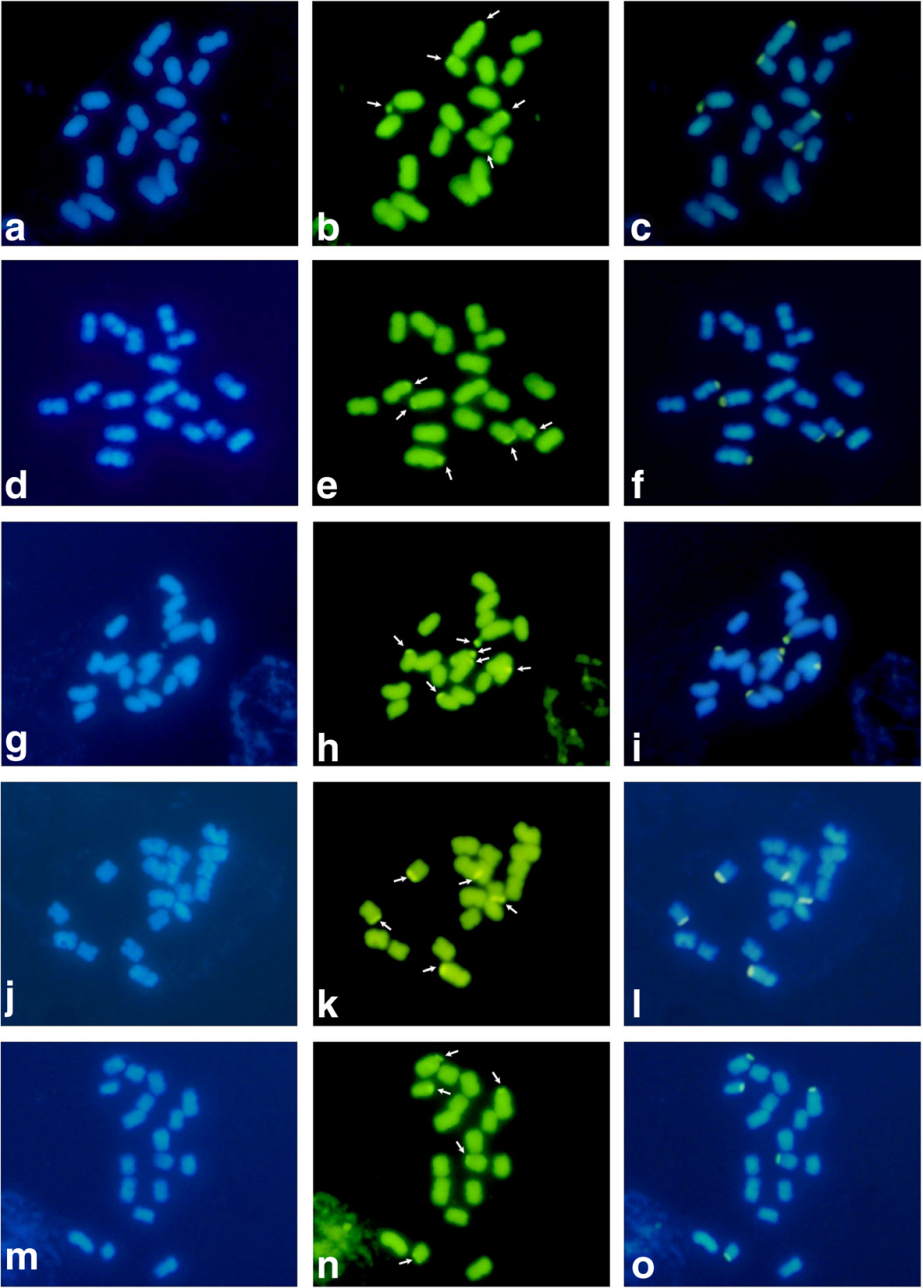
CMA_3_/DA/DAPI banding of mitotic metaphase cells from interspecific hybrids of *Passiflora* HD15 progeny. Staining with DAPI (**a**, **d**, **g**, **j**, **m**), CMA_3_ (**b**, **e**, **h**, **k**, **n**) and CMA_3_/DAPI merged (**c**, **f**, **i**, **l**, **o**). **a**-**c**: HD15-107; **d**-**f**: HD15-108; **g**-**i**: HD115-109; **j**-**l**: HD15-110; **m**-**o**: HD15-111. Arrows indicate CMA_3_^+^ blocks. Bar = 10 μm

Here, conventional staining was only able to aid in counting chromosome number (*2n* = 18; Additional file 1). CMA_3_/DA/DAPI banding permitted the observation of satellites (secondary constriction) not visible with conventional staining. No DAPI^+^ blocks were observed, and CMA_3_^+^/DAPI^−^ blocks were restricted to satellites and secondary constrictions (Figs. 1, 2, and 5). The relationship between the CMA_3_^+^/DAPI^−^ terminal blocks and satellites (secondary constriction) allowed for the confirmation of the number of satellites (secondary constriction) in both genitor species. Six CMA_3_^+^/DAPI^−^ blocks were observed in the maternal parent (*P. gardneri*) and five in the paternal parent (*P. gibertii*). In the same individual analyzed, it was also observed a heteromorphic pair after conventional staining, with a single homolog carrying a satellite (secondary constriction) (Table 1). It was possible to observe CMA_3_^+^ blocks, confirming the number of satellites (secondary constriction) in the eight analyzed hybrids (Table 1).

**Table 1:** Karyotypic data based on CMA_3_/DA/DAPI banding and FISH in *Passiflora* parents and interspecific hybrids

Genotype	CMA_3_^+^	45S rDNA	5S rDNA
*P. gardneri*	6	6	4
*P. gibertii*	5	5	2
HD15-101	5	5 (3 M; 2P)	3 (2 M; 1P)
HD15-104	5	5 (3 M; 2P)	3 (2 M; 1P)
HD15-106	6	6 (3 M; 3P)	3 (2 M; 1P)
HD15-107	5	5 (3 M; 2P)	3 (2 M; 1P)
HD15-108	5	5 (3 M; 2P)	3 (2 M; 1P)
HD15-109	6	6 (3 M; 3P)	3 (2 M; 1P)
HD15-110	5	5 (3 M; 2P)	3 (2 M; 1P)
HD15-111	5	5 (3 M; 2P)	3 (2 M; 1P)

*CMA*_*3*_^*+*^ number of CMA_3_^+^ blocks, *45S rDNA* number of 45S rDNA sites, *5S rDNA* number of 5S rDNA sites. *M* site of maternal origin, *P* site of paternal origin

### Gish

To check the relationship between the amount of blocking DNA and the probe, it is necessary to adjust blocking DNA concentrations to distinguish genomes. In this study, it was necessary to use 100× more blocking DNA than the probe to identify putative hybrids. No satisfactory results were obtained when using lower concentrations of blocking DNA, likely owing to strong cross-hybridization with the non-target genome.

**Fig. 3:**
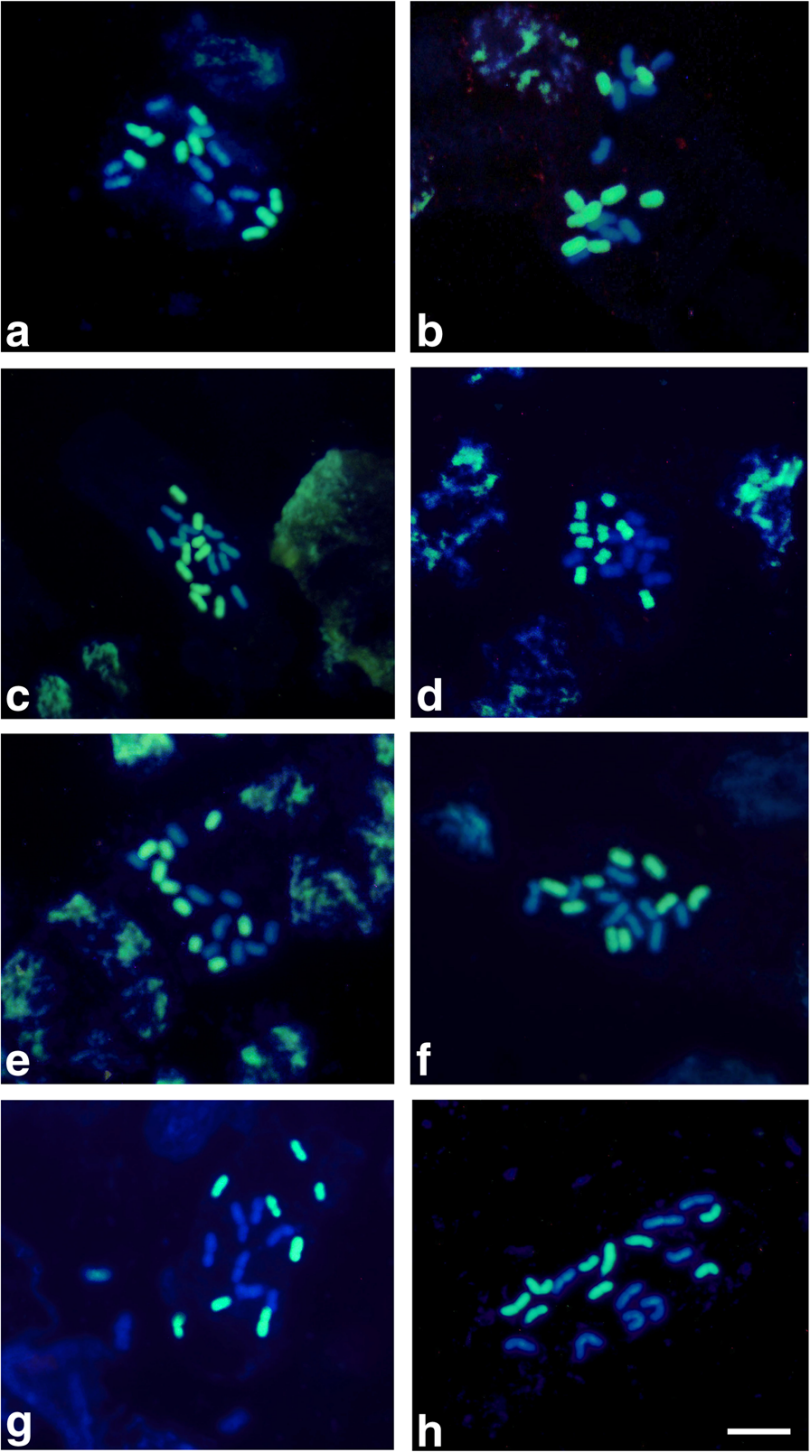
Genomic in situ hybridization (GISH) analysis of mitotic metaphase cells from interspecific hybrids of *Passiflora* HD15 progeny. **a** HD15-101, (**b**) HD15-104, (**c**) HD15-106, (**d**) HD15-107, (**e**) HD15-108, (**f**) HD15-109, (**g**) HD15-110, (**h**) HD15-111. Bar = 10 μm

GISH distinguished each parental chromosome set within the analyzed hybrids. In each plant, the nine chromosomes from the paternal parent were uniformly and wholly labeled with FITC, while the remaining nine chromosomes of maternal origin were unlabeled or presented a very low level of signal due to cross-hybridization (DAPI counterstaining). Hybrids, like their parents, must be diploid individuals possessing 2*n* = 18 chromosomes. GISH confirmed the hybrid character in all analyzed HD15 progeny plants (Fig. 3).

### 45S and 5S rDNA FISH

**Fig. 4:**
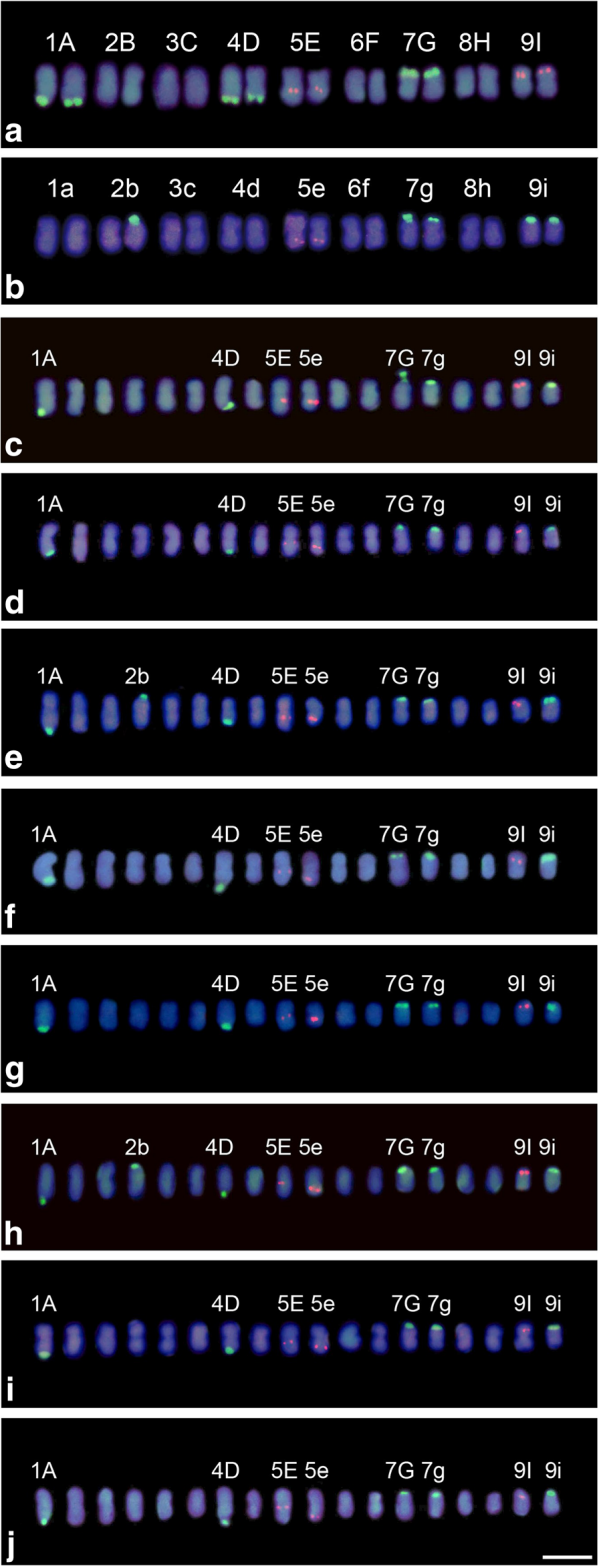
Karyograms with 5S and 45S rDNA probes for parents and interspecific hybrids of *Passiflora* HD15 progeny. **a** *P. gardneri* Mast., (**b**) *P. gibertii* N. E. Brown, (**c**) HD15-101, (**d**) HD15-104, (**e**) HD15-106, (**f**) HD15-107, (**g**) HD15-108, (**h**) HD15-109, (**i**) HD15-110, (**j**) HD15-111. Letters and numbers for parent karyograms indicate chromosome pairs. Letters and numbers for hybrid karyograms indicate chromosomes with 45S and 5S rDNA sites. Bar = 10 μm

**Fig. 5:**
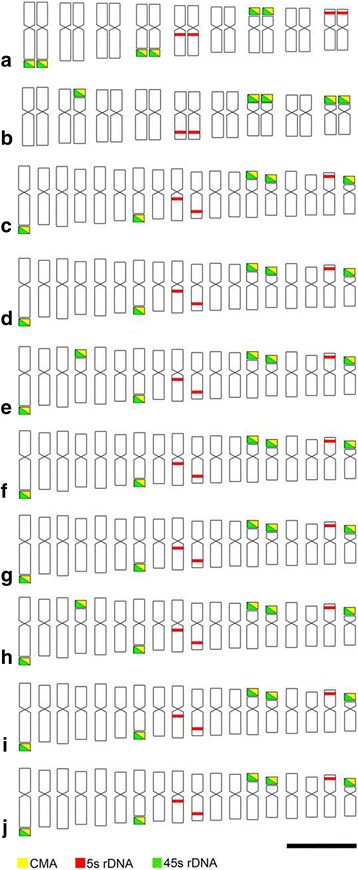
Ideograms showing CMA_3_ blocks and 5S and 45S rDNA sites in parents and interspecific hybrids of *Passiflora* HD15 progeny. **a** *P. gardneri* Mast., (**b**) *P. gibertii* N. E. Brown, (**c**) HD15-101, (**d**) HD15-104, (**e**) HD15-106, (**f**) HD15-107, (**g**) HD15-108, (**h**) HD15-109, (**i**) HD15-110, (**j**) HD15-111. Bar = 5 μm

The 45S and 5S rDNA sites were mapped in both parent plants and the eight interspecific hybrids (HD15) (Figs. 4 and 5). The number of 45S and 5S rDNA sites within each hybrid, as well as their parental origin, are shown in Table 1.

Parental karyotype identification was performed as follows: chromosome pairs were ordered by size in descending order, with *P. gardneri* chromosomes named 1A to 9I and *P. gibertii* chromosomes named 1a to 9i. Hybrid genotype karyotype denomination was carried out by identifying parental chromosome markers using 45S and 5S rDNA hybridization sites, which were segregated in the hybrid progeny HD15. Chromosome pairs 1A, 4D, and 7G for *P. gardneri* presented 45S rDNA sites, while chromosome pairs 5E and 9I presented 5S rDNA sites. In *P. gibertii*, chromosome pairs 2b, 7 g, and 9i presented 45S rDNA sites, while pair 5e presented 5S rDNA sites (Fig. 4).

Hybrid karyotype analyses were based on the presence of marker chromosomes. The chromosomes with 45S and 5S rDNA sites maintained the same positions as in the genitor species. To facilitate identification, only marker chromosomes were numbered and named in the karyograms of the eight analyzed hybrids (Fig. 4c-j).

For the maternal genome (*P. gardneri*), chromosome 1A, which has a 45S rDNA site on the long arm, was chosen as the primary marker identifying the presence of this genome in the hybrid because no hybridization signal from this chromosome was found in the paternal genome. Only the maternal genome was found to have 45S rDNA sites in chromosomal long arms. Moreover, the fact that chromosome 1A is longer than the others offers a uniqueness that prevents confusion. Chromosome 5E, which is unique in having a 5S rDNA site in the pericentromeric region of the long arm, was used as a secondary marker.

For the paternal genome (*P. gibertii*), chromosome 5e, which has a 5S rDNA site in the terminal region of the long arm, was used as the primary marker, because this characteristic is exclusive for the paternal genome. Chromosome 9i, with a 45S rDNA site in the terminal region of the short arm, was used as secondary marker, since it was the smallest chromosome present in the hybrids. The other chromosomes presenting rDNA sites could not be used as identifying markers in maternal and paternal genome due to site and size similarities.

The eight analyzed hybrids presented chromosomes with 45S and 5S rDNA sites in the characteristic positions aligning with each donor genome. In hybrids HD15-101, HD15-104, HD15-107, HD15-108, HD15-110, and HD15-111, five 45S rDNA sites and three 5S rDNA sites were clearly observed, while six 45S rDNA sites and three 5S rDNA sites were found in hybrids HD15-106 and HD15-109 (Table 1). For all analyzed plants, hybridization was confirmed through the presence of genome marker chromosomes.

## Discussion

Interspecific hybridization has been conducted in *Passiflora* mainly for the production of new ornamental varieties with more attractive flowers and colors. The methods used for hybrid identification within the genus are mainly based on morphological characteristics [9], as well as the usage of RAPD [18, 19] and SSR [8] molecular markers. The application of classic, banding, and molecular cytogenetic techniques can be useful in hybrid identification, karyological characterization, chromosome stability analysis, and hybrid selection for breeding programs.

Karyotype analysis using only classical cytogenetic methods for hybrid identification was not possible due to the very similar morphologies between the chromosomes and difficulties in visualizing the satellites (secondary constrictions) using Giemsa staining alone. Unclear Giemsa staining results could lead to inaccurate hybrid identification. In a survey done in 2005, it was found that in most species of *Passiflora*, the utility of karyotype characterization was restricted to counting the number of chromosomes [32]. The lack of karyomorphologic data for many species and generated hybrids within the genus is likely due to karyotype similarity [33]. However, we observed chromosome stability, as a constant diploid number of chromosomes was found in all hybrid germplasms investigated, as well as in the genitor species. The absence of chromosome elimination or disploidy is a positive attribute for potential breeding, as disploidy could present reproductive and fertilization issues, and species bearing this phenomenon are not recommended for use as genetic resources in breeding programs.

The detection of GC- and AT-rich heterochromatin regions can assist in hybrid characterization. CMA_3_/DAPI banding was used to verify GC-rich (CMA_3_^+^) and AT-rich (DAPI^+^) regions. Here, GC-rich regions were restricted to the satellites (secondary constrictions), while AT-rich regions were not directly visible (identified instead by DAPI^−^ regions co-located with GC-rich regions. These results corroborated what has been previously described in other species of the genus *Passiflora* [22, 26, 34]. In our study, the absence of CMA^+^/DAPI^−^ blocks in some hybrids was possibly due to the presence of a heteromorphic pair in the paternal parent (*P. gibertii*). This difference in the number of satellites between F_1_ hybrids could lead to chromosomal changes in F_2_ hybrids caused by unequal recombination during meiosis. This hypothesis could be further examined via a meiotic study or by cytological analysis of F_2_ hybrids using 45S rDNA probes or other specific chromosomal markers.

GISH is an efficient method for hybrid identification because it allows the determination of chromosomal genomic origin even without previous knowledge of chromosome morphology [12, 16]. It also allows the observation of recombination or alterations between different genomes [35]. In this study, GISH was successfully used to confirm hybrid status and no chromosome translocation was found. The optimization of GISH conditions allowed for the uniform labeling of all paternal-origin chromosomes and minimal cross-hybridization signal from maternal-origin chromosomes. Optimal results were obtained when blocking DNA was used at a 100× higher concentration relative to the probe. The need for such a high blocking DNA concentration suggests that both parents share many repetitive DNA sequences, which was understandable given the close taxonomic relationship between the genitor species [36]. It was thus necessary to adjust the amount of blocking DNA used in accordance with the amount of sequence DNA shared between the species used for crossing [37]. In an F_1_ hybrid obtained between two species of great economic and agronomic interest (*P. edulis* vs. *P. cincinnata*), it was not possible to identify complete chromosome subsets (nine chromosomes) specific to each parental species. Instead, three chromosome subsets were identified: eight chromosomes from *P. edulis* (completely labeled by the probe), six partially labeled chromosomes, and four unlabeled chromosomes. These results were likely due to the use of a low concentration of blocking DNA, since the partial hybridization of some chromosomes may have occurred because the parental species were phylogenetically related and share significant amounts of DNA sequences [38]. Conversely, an investigation of F_1_ and RC_1_ hybrids involving the species *P. sublanceolata* (Genoma-S) and *P. foetida* (Genoma-F) was able to identify and confirm hybrid status and visualize chromosomal recombination in RC_1_ hybrids and elucidation of triploidy origin in a RC_1_ hybrid [21]. These results demonstrate the successful occurrence of chromosomal recombination among different *Passiflora* species, indicating hybrid generation potential.

In this study, rDNA was demonstrated to be useful for identifying hybrid status, as well as determining chromosomal stability through analysis of the number and localization of chromosomal markers. The presence of stable karyotypes in hybrids allows useful plants to be selected and breeding programs to be advanced. Although both genitor species had metacentric and similarly sized chromosomes, chromosome-specific 45S and 5S rDNA probe-labeling provided chromosome markers with unique characteristics for each parent species, and thus allowed the reliable confirmation of hybrid status. FISH techniques using two or more repetitive DNA sequences as probes have been widely used for chromosome identification, and consequently have been able to serve as chromosome markers in certain plant species such as those of the genus *Lilium* L. [12]. The simultaneous use of 45S and 5S rDNA probes provided chromosome markers that were used for the identification of genomic material from each donor, and thereby facilitated determination of the hybrid status of *Lilium* [12]. In the genus *Oryza* L., the application of 45S rDNA probes in hybrids (*O. meyriana* vs. *O. sativa*) identified two 45S rDNA sites belonging to *O. meyriana* and one site belonging to *O. sativa* [39]. In *Passiflora*, among the nine pairs of chromosomes of each parent species, four pairs – two maternal and two paternal – could be used as markers.

The variation in the amount of 45S rDNA sites in the hybrids analyzed in this study is due to the paternal genitor species presenting heteromorphic chromosome pair 2b, which only presents a 45S rDNA site in one homolog. Thus, during meiosis this species may form gametes containing either two or three chromosomes carrying 45S rDNA sites. In hybrids containing five 45S rDNA sites, there was a fusion of a paternal gamete carrying two 45S rDNA sites with a maternal gamete carrying three 45S rDNA sites, whereas in hybrids containing six 45S rDNA sites, there was a fusion of a paternal gamete carrying three 45S rDNA sites with a maternal gamete carrying three 45S rDNA sites. The presence of a heteromorphic homologous chromosome pair in *P. gibertii* was probably due to 45S rDNA site deletion or reduction, which could not be detected using FISH on chromosomes in metaphase. Alternatively, this species presented individual differences, with some individuals carrying four 45S sites and others carrying six 45S sites. Crossing between these different individuals could result in individuals with five 45S rDNA sites.

## Conclusions

Karyotype data obtained in this study showed that the hybrids are cytologically stable. FISH demonstrated that the simultaneous use of rDNA probes provided unique chromosome markers from each parent, facilitating the recognition of each genome genitor in the hybrids, consequently confirming paternity. Similarly, GISH was successfully used for hybrid status confirmation. The application of GISH is poorly explored for the purpose of improving *Passiflora* species, and thus, technique optimization and the results from this study will contribute to the improvement of breeding programs involving species from this genus. Besides hybridization confirmation, GISH also allows the visualization of recombination between the homeologous chromosome and the introgression of sequences of interest.

## Additional file

Additional file 1: Giemsa staining of mitotic metaphase cells from parents and interspecific hybrids of *Passiflora* HD15 progeny (2*n* = 18). (A) *P. gardneri* Mast., (B) *P. gibertii* N. E. Brown, (C) HD15-101, (D) HD15-104, (E) HD15-106, (F) HD15-107, (G) HD15-108, (H) HD15-109, (I) HD15-110, (J) HD15-111. Bar = 10 μm. (TIFF 3954 kb)

## Abbreviations

2n: Diploid number
8-HQ: 8-hydroxyquinoline
AFLP: Amplified fragment length polymorphism
BSA: Bovine Serum Albumin
CMA_3_: Chromomycin A_3_
DA: Distamycin A
DAPI: 4′-6-diamidino-2-phenylindole
ESTs: Expressed sequence tags
FISH: Fluorescence in situ hybridization
FITC: Fluorescein isothiocyanate
GISH: genomic in situ hybridization
HCl: Hydrochloric acid
PCR: Polymerase chain reaction
RAPD: Random amplified polymorphic DNA
rDNA: ribosomal DNA
RT: Room temperature
SDS: Sodium dodecyl sulfate
SSC: Salt, sodium citrate
SSR: Simple sequence repeat
