# Supplementary figures and images for: Identification and characterization of karyotype in Passiflora hybrids using FISH and GISH
Source: BMC Genet. 2018 Apr 27;19:26. doi: 10.1186/s12863-018-0612-0 (PMC5921547; doi:10.1186/s12863-018-0612-0)

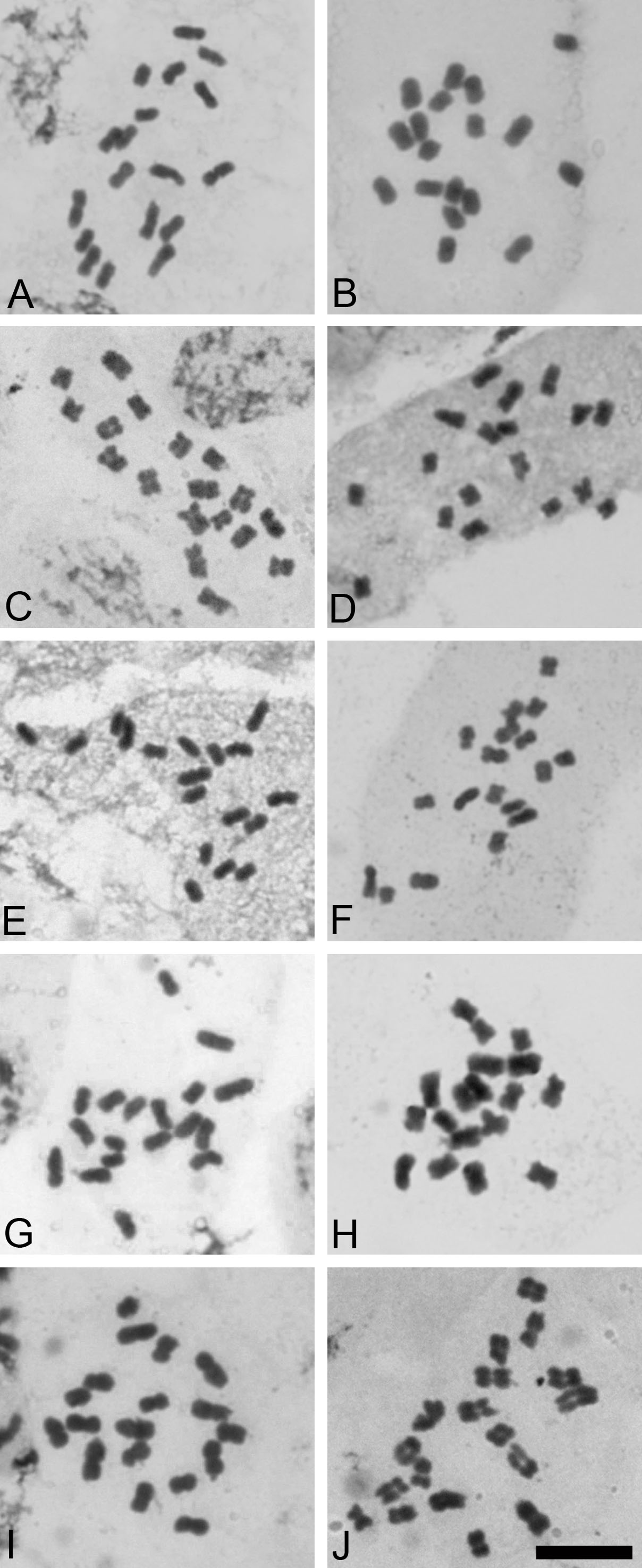

Supplement: Supplementary file 1 — Giemsa staining of mitotic metaphase cells from parents and interspecific hybrids of Passiflora HD15 progeny (2n = 18). (A) P. gardneri Mast., (B) P. gibertii N. E. Brown, (C) HD15-101, (D) HD15-104, (E) HD15-106, (F) HD15-107, (G) HD15-108, (H) HD15-109, (I) HD15-110, (J) HD15-111. Bar = 10 μm. (TIFF 3954 kb) [file 12863_2018_612_MOESM1_ESM.tif]
